# Supplementary material for: A self-amplifying RNA RSV prefusion-F vaccine elicits potent immunity in pre-exposed and naïve non-human primates
Source: Nat Commun. 2024 Nov 14;15:9884. doi: 10.1038/s41467-024-54289-9 (PMC11564874; doi:10.1038/s41467-024-54289-9)
Supplement: Supplementary file 1 — Supplementary Information [file 41467_2024_54289_MOESM1_ESM.pdf]

Material	Test	Assay	Result
saRNA	Integrity	Fragment analyzer	76% full length
	Potency	dsRNA launch assay	95%
	Residual protein	NanoOrange assay	3.7E-05 mg/mg RNA
	Endotoxin	LAL assay	<2 EU/mg RNA
LNP forumulated saRNA	Size	DLS assay	75±5 nm
	PDI	DLS assay	0.14
	Encapsulation efficiency	Ribogreen assay	99%
	Endotoxin	LAL assay	<2 EU/mg RNA

Supplementary Table 1 | Quality test results of SMARRT.RSV.preF and LNP formulated material.

Hallmark gene set	FDR	Analyte	Linear FC	Adjusted p.value
Interferon Gamma response	0.0128	CCL2	3.65	3.17E-11
		CXCL10	9.28	2.86E-18
		CXCL11	8.52	3.51E-15
		CXCL9	2	5.06E-04
		IL15	3.73	6.34E-16
		IL6	11.4	1.08E-07
		IL7	1.18	2.72E-01
		TNFSF10	1.69	2.47E-06
Interferon Alpha response	0.0324	CSF1	1.53	2.01E-08
		CXCL10	9.28	2.86E-18
		CXCL11	8.52	3.51E-15
		IL15	3.73	6.34E-16
		IL7	1.18	2.72E-01
TNFα signaling via NFκB	0.0372	OLR1	1.02	8.76E-01
		IL1β	0.836	2.44E-01
		IL6	11.4	1.08E-07
		IL18	1.64	2.68E-04
		CXCL10	9.28	2.86E-18
		TNF	1.76	1.64E-08
		VEGFA	1.47	1.22E-06
		CCL2	3.65	3.17E-11
		CSF1	1.53	2.01E-08
		CCL4	1.4	3.18E-02
		CXCL11	8.52	3.51E-15
Inflammatory response	0.0372	OLR1	1.02	8.76E-01
		IL1β	0.836	2.44E-01
		IL6	11.4	1.08E-07
		IL18	1.64	2.68E-04
		TNFSF10	1.69	2.47E-06
		CXCL10	9.28	2.86E-18
		IL15	3.73	6.34E-16
		CXCL8	1.14	3.19E-01
		CCL2	3.65	3.17E-11
		OSM	1.33	8.12E-02
		CSF1	1.53	2.01E-08
		CXCL11	8.52	3.51E-15
		LTA	1.01	8.76E-01
		CXCL9	2	5.06E-04

Supplementary Table 2 | Biological processes influenced by systemic cytokines/chemokines differentially regulated by immunization with 10mcg of SMARRT.RSV.preF. Analytes related to biological processes were identified by performing a gene set enrichment analysis using the camera algorithm using the MSigDB Hallmark gene set collection (n = 8 biologically independent animals). Gene sets with false discovery rate (FDR) adjusted p.values<0.05 (moderated two-sided t-statistics) are shown. Analytes that are part of each gene set are shown. For each analyte the estimated mean fold change (on a linear scale) and associated adjusted p.value (two-sided moderated t-test) as obtained from fitting a linear model with time (pre- to post- immunization) as factor is also shown.

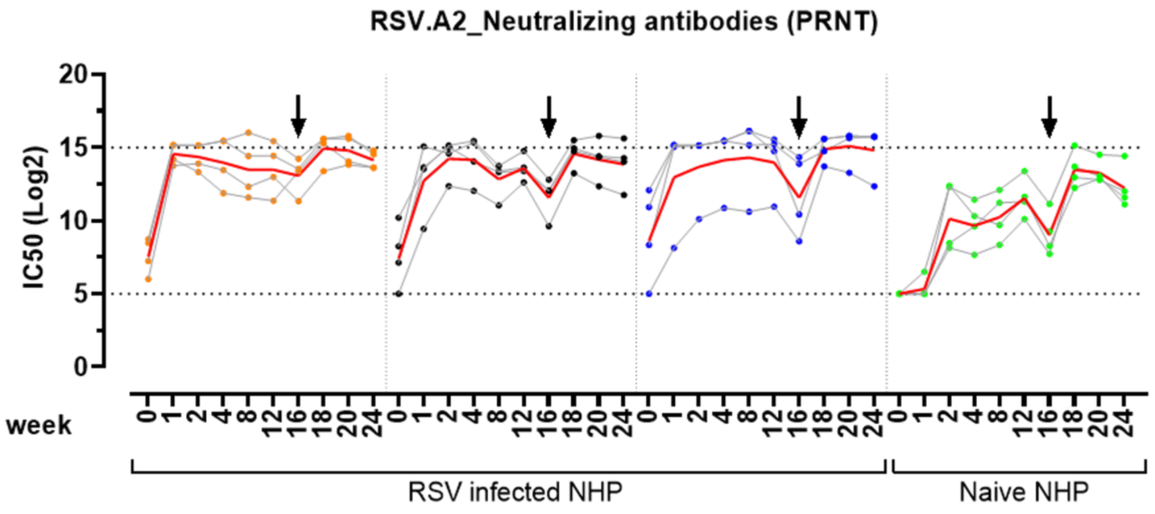

Legend	1st dose (week 0)		Booster (week 16)	
	Vaccine	Dose (mcg)	Vaccine	Dose (mcg)
●	PRPM	50	PRPM	50
●	SMARRT.RSV.preF	1	SMARRT.RSV.preF	1
●	SMARRT.RSV.preF	10	SMARRT.RSV.preF	1
●	SMARRT.RSV.preF	10	SMARRT.RSV.preF	1

Supplementary Fig. 1 | RSV.A2 PRNT measured in the serum of immunized animals. Lower and upper limit of quantification indicated by the dotted lines. Individual animals (n = 4/group) are represented by the dots color coded according to treatment with geometric mean response by the red line. Animals were boosted at week 16 indicated by the down arrow. No statistical analysis was done since the majority of RSV pre-exposed animals had PRNT at ULoQ.

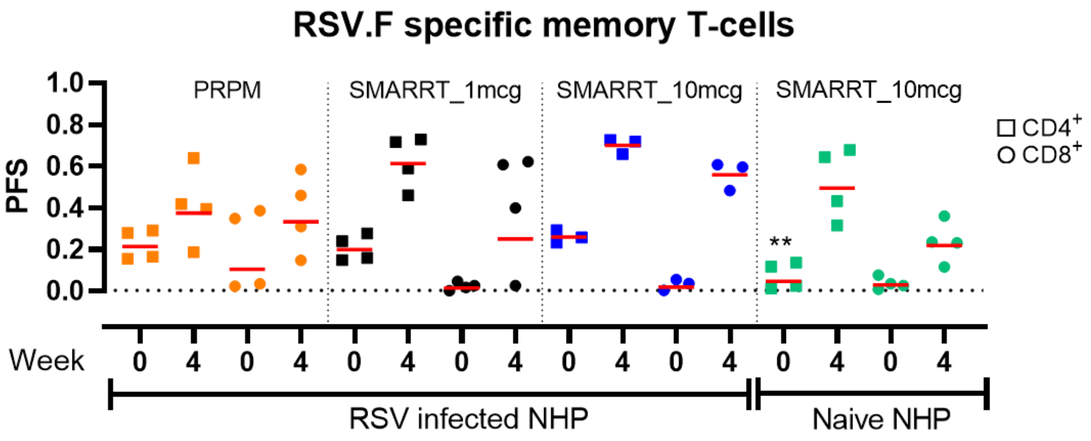

Supplementary Fig. 2 | COMPASS polyfunctionality score (PFS) for RSV.F specific memory CD4 (squares) and CD8 (circles) T-cells were determined at week 0 (pre-immunization) and at week 4 (*n* = 4/group). Due to insufficient number of cells, data from an RSV pre-exposed animal immunized with 10mcg SMARRT.RSV.preF group is not available. Statistical significance for CD4 and CD8 PFS was estimated with ANOVA by comparing the response between SMARRT immunized groups at weeks 0 and at week 4. ** p<0.01.

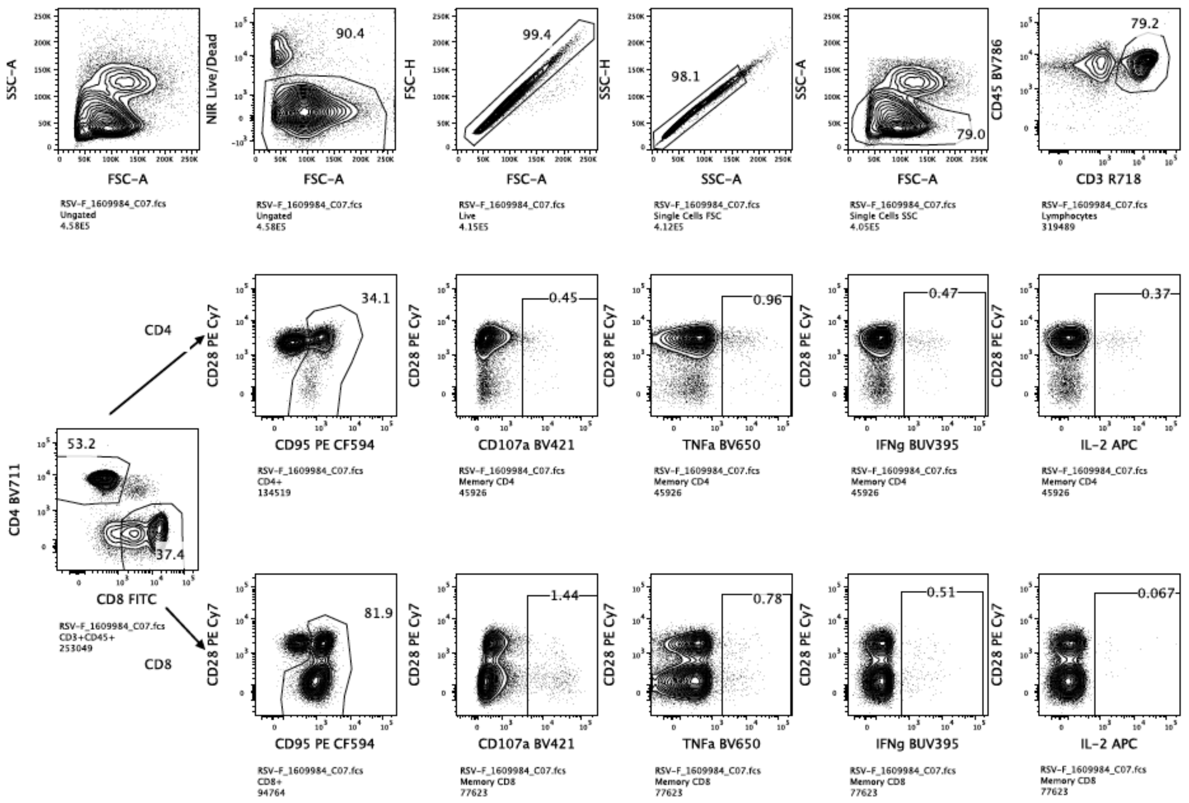

Supplementary Fig. 3 | Gating strategy for intracellular cytokine staining of PBMCs from NHPs.

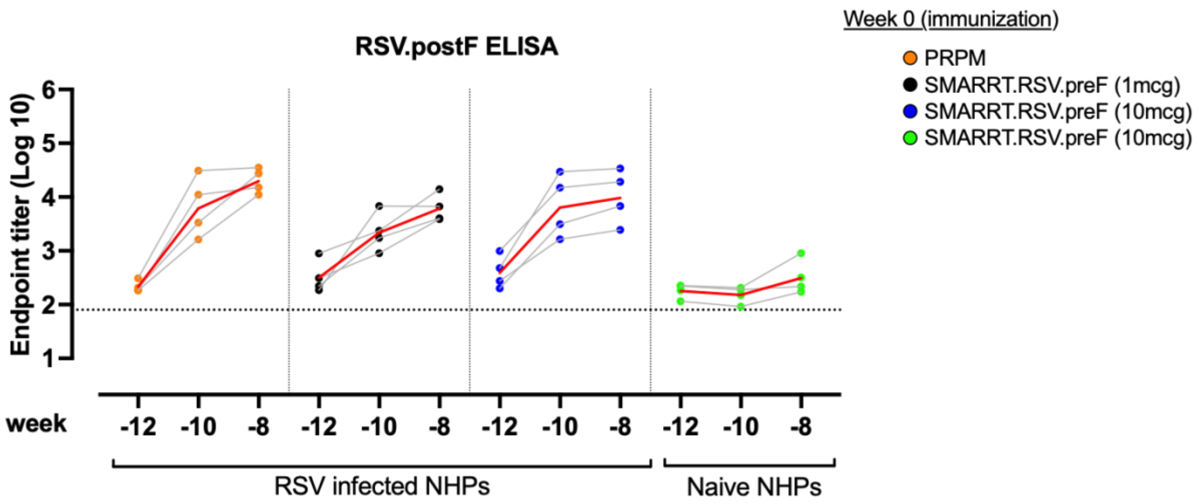

Supplementary Fig. 4 | Binding antibody titers specific to RSV post-F protein were measured in the serum of NHPs following RSV infection at week -12. The figure displays the temporal kinetics of antibody titers in RSV infected animals (n = 12) that were distributed into three groups (n = 4/group). A naïve group (n = 4) was not infected with RSV (green). The limit of detection (LoD) is represented by the dotted line, and the geometric mean response is shown by the red line. The animals in respective groups were immunized with vaccines at week 0.
