# Supplementary material for: A self-amplifying RNA RSV prefusion-F vaccine elicits potent immunity in pre-exposed and naïve non-human primates
Source: Nat Commun. 2024 Nov 14;15:9884. doi: 10.1038/s41467-024-54289-9 (PMC11564874; doi:10.1038/s41467-024-54289-9)
Supplement: Supplementary file 2 — Reporting Summary [file 41467_2024_54289_MOESM2_ESM.pdf]

nature portfolio

Corresponding author(s): Aneesh Vijayan, Roland Zahn

Last updated by author(s): Oct 29, 2024

Reporting Summary

Nature Portfolio wishes to improve the reproducibility of the work that we publish. This form provides structure for consistency and transparency in reporting. For further information on Nature Portfolio policies, see our Editorial Policies and the Editorial Policy Checklist.

Statistics

For all statistical analyses, confirm that the following items are present in the figure legend, table legend, main text, or Methods section.

n/a	Confirmed	
☐	☒	The exact sample size (*n*) for each experimental group/condition, given as a discrete number and unit of measurement
☐	☒	A statement on whether measurements were taken from distinct samples or whether the same sample was measured repeatedly
☐	☒	The statistical test(s) used AND whether they are one- or two-sided<br>*Only common tests should be described solely by name; describe more complex techniques in the Methods section.*
☐	☒	A description of all covariates tested
☐	☒	A description of any assumptions or corrections, such as tests of normality and adjustment for multiple comparisons
☐	☒	A full description of the statistical parameters including central tendency (e.g. means) or other basic estimates (e.g. regression coefficient) AND variation (e.g. standard deviation) or associated estimates of uncertainty (e.g. confidence intervals)
☐	☒	For null hypothesis testing, the test statistic (e.g. *F*, *t*, *r*) with confidence intervals, effect sizes, degrees of freedom and *P* value noted<br>*Give P values as exact values whenever suitable.*
☒	☐	For Bayesian analysis, information on the choice of priors and Markov chain Monte Carlo settings
☒	☐	For hierarchical and complex designs, identification of the appropriate level for tests and full reporting of outcomes
☒	☐	Estimates of effect sizes (e.g. Cohen's *d*, Pearson's *r*), indicating how they were calculated

Our web collection on statistics for biologists contains articles on many of the points above.

Software and code

Policy information about availability of computer code

Data collection	ELISA and VNA luminescence readout was obtained in the Gen5™ Data Analysis Software.<br>Flow cytometry data was collected with BD FACSDiva software v.9.0.<br>Serum cytokine profiling was obtained with Olink NPX™ Software.
Data analysis	Gen5™ Data Analysis Software for estimating ELISA and VNA titers.<br>FlowJo software v.9.6.1 was used for flow cytometry data analysis.<br>SPICE 6.0 and COMPASS ver 1.17.0 were used to determine polyfunctionality of T-cells.<br>Graph Pad Prism versions 9.0.0 and 9.5.0 for plotting graphs.<br>R ver 4.12, Limma Ver 3.50.3, Corrplot ver 0.92 and Msigdbr ver 7.5.1 were used to analyze Serum cytokine data

For manuscripts utilizing custom algorithms or software that are central to the research but not yet described in published literature, software must be made available to editors and reviewers. We strongly encourage code deposition in a community repository (e.g. GitHub). See the Nature Portfolio guidelines for submitting code & software for further information.

Data

Policy information about availability of data

All manuscripts must include a data availability statement. This statement should provide the following information, where applicable:

- Accession codes, unique identifiers, or web links for publicly available datasets
- A description of any restrictions on data availability
- For clinical datasets or third party data, please ensure that the statement adheres to our policy

All of the final data has been included in main figures or supplementary information. Any requests for protocols and reagents should be directed to the corresponding authors to be fulfilled under reasonable request.

Research involving human participants, their data, or biological material

Policy information about studies with human participants or human data. See also policy information about sex, gender (identity/presentation), and sexual orientation and race, ethnicity and racism.

Reporting on sex and gender	N/A
Reporting on race, ethnicity, or other socially relevant groupings	N/A
Population characteristics	N/A
Recruitment	N/A
Ethics oversight	N/A

Note that full information on the approval of the study protocol must also be provided in the manuscript.

Field-specific reporting

Please select the one below that is the best fit for your research. If you are not sure, read the appropriate sections before making your selection.

☒ Life sciences ☐ Behavioural & social sciences ☐ Ecological, evolutionary & environmental sciences

For a reference copy of the document with all sections, see nature.com/documents/nr-reporting-summary-flat.pdf

Life sciences study design

All studies must disclose on these points even when the disclosure is negative.

Sample size	Sample size of the mouse and NHP studies described in the manuscript are determined based on power calculations.<br>Historical data for the investigated or similar vaccines were used to guide statistical powering. Sample sizes were calculated using SAS version 9.4 (SAS Institute, Inc., Cary, NC, USA)
Data exclusions	No data excluded. Due to insufficient number of cells, no flow cytometry data available for one NHP from an RSV infected group immunized with 10mcg of SMARRT.RSV.preF.
Replication	Virologic and immunologic measures were performed in duplicate and once. Technical replicates were minimally different.
Randomization	Animals were balanced for age and gender and otherwise randomly allocated to groups.
Blinding	Investigators were blinded during data collection. For humoral assays, serum samples were randomly allocated onto the measurement plates to minimize group specific bias.

Reporting for specific materials, systems and methods

We require information from authors about some types of materials, experimental systems and methods used in many studies. Here, indicate whether each material, system or method listed is relevant to your study. If you are not sure if a list item applies to your research, read the appropriate section before selecting a response.

Materials & experimental systems

n/a	Involved in the study
☐	☒ Antibodies
☐	☒ Eukaryotic cell lines
☒	☐ Palaeontology and archaeology
☐	☒ Animals and other organisms
☒	☐ Clinical data
☒	☐ Dual use research of concern
☒	☐ Plants

Methods

n/a	Involved in the study
☒	☐ ChIP-seq
☐	☒ Flow cytometry
☒	☐ MRI-based neuroimaging

Antibodies

Antibodies used	All antibodies and associated information have been listed in the supplemental material.
Validation	All mAbs were used according to manufacturer's instructions and previously published methods; mAbs were validated and titrated for specificity prior to use

Eukaryotic cell lines

Policy information about cell lines and Sex and Gender in Research

Cell line source(s)	BHK and A549 cells were commercially purchased (ATCC)
Authentication	All cell lines sourced from ATCC and validated by ATCC
Mycoplasma contamination	Negative for mycoplasma
Commonly misidentified lines (See ICLAC register)	Not used

Animals and other research organisms

Policy information about studies involving animals; ARRIVE guidelines recommended for reporting animal research, and Sex and Gender in Research

Laboratory animals	Mouse study: BALB/c mice female, 6 to 8 weeks old at the start of the study<br>NHP study: Cynomolgous macaques, 6 to 7 years old (6 male and 6 female)
Wild animals	None
Reporting on sex	Mouse study were performed only with female animals.<br>For NHP study animals were randomly allocated to have similar number of males and females in each group.
Field-collected samples	N/A
Ethics oversight	The mouse studies at Janssen Vaccines and Prevention B.V. were conducted in accordance with the Dutch Animal Experimentation Act and the Guidelines on the Protection of Animals for scientific purposes by the Council of the European Committee. These studies were approved by the Centrale Commissie Dierproeven and the Dier Experimenten Commissie of Janssen Vaccines and Prevention B.V. Additionally, the NHP study was conducted at the Alpha Genesis test facility and was approved by the IACUC committee of Alpha Genesis (IACUC#22-11).

Note that full information on the approval of the study protocol must also be provided in the manuscript.

Plants

Seed stocks	N/A
Novel plant genotypes	N/A
Authentication	N/A

Flow Cytometry

Plots

Confirm that:

☒ The axis labels state the marker and fluorochrome used (e.g. CD4-FITC).

☒ The axis scales are clearly visible. Include numbers along axes only for bottom left plot of group (a 'group' is an analysis of identical markers).

☒ All plots are contour plots with outliers or pseudocolor plots.

☒ A numerical value for number of cells or percentage (with statistics) is provided.

Methodology

Sample preparation	10^6 PBMCs/well were re-suspended in 100 µL of R10 media
Instrument	FACS Canto II instrument (BD Biosciences)
Software	FlowJo ver9.6.1
Cell population abundance	N/A
Gating strategy	See supplementary figure 3 (Fig S3)

☒ Tick this box to confirm that a figure exemplifying the gating strategy is provided in the Supplementary Information.
